# Association between weight-adjusted waist index and cardiometabolic multimorbidity in older adults: Findings from the English Longitudinal Study of Ageing

**DOI:** 10.1007/s11357-025-01829-w

**Published:** 2025-08-07

**Authors:** Setor K. Kunutsor, Sae Young Jae, Jari A. Laukkanen

**Affiliations:** 1 Section of Cardiology, Department of Internal Medicine, Rady Faculty of Health Sciences, Max Rady College of Medicine, University of Manitoba, Winnipeg, MB R2H 2A6 Canada; 2 Leicester Real World Evidence Unit, Diabetes Research Centre, University of Leicester, Leicester General Hospital, Gwendolen Road, Leicester, LE5 4WP UK; 3 Department of Sport Science, University of Seoul, Seoul, Republic of Korea; 4 Institute of Clinical Medicine, Department of Medicine, University of Eastern Finland, Kuopio, Finland; 5 Department of Medicine, Wellbeing Services County of Central Finland, Jyväskylä, Finland

**Keywords:** Weight-adjusted waist index, Visceral adiposity, Cardiometabolic multimorbidity, Cohort study

## Abstract

The weight-adjusted waist index (WWI) is a novel anthropometric measure designed to better reflect central obesity than traditional indices such as body mass index and waist circumference (WC). This study examined the prospective association between WWI and cardiometabolic multimorbidity (CMM) and evaluated its predictive utility. We included 3,348 participants (mean age 63 years; 45.1% male) from the English Longitudinal Study of Ageing who were free from hypertension, coronary heart disease, diabetes, and stroke at baseline (wave 4: 2008–2009). WWI was calculated as WC (cm) divided by the square root of body weight (kg). CMM was defined as the presence of ≥ 2 of the following conditions at wave 10 (2021–2023): hypertension, cardiovascular disease, diabetes, or stroke. Multivariable logistic regression and measures of discrimination were used to assess associations and predictive value. Over 15 years, 197 participants developed CMM. Restricted cubic spline analysis indicated a linear dose–response relationship between WWI and CMM risk (*p* for nonlinearity = .44). Each 1 SD increase in WWI was associated with higher odds of CMM (odds ratio, OR = 1.30; 95% CI: 1.12–1.51), persisting after adjustment for physical activity (OR = 1.28; 95% CI: 1.10–1.49). Similar associations were observed across WWI tertiles. Adding WWI to conventional risk models slightly improved discrimination (ΔC-index = 0.0065; *p* = .29), with a significant improvement in model fit (-2 log likelihood, p = .001). Higher WWI levels were independently and linearly associated with increased CMM risk in older adults. WWI also improved CMM risk prediction beyond conventional risk factors.

**Supplementary Information:**

The online version contains supplementary material available at 10.1007/s11357-025-01829-w.

## Introduction

The presence of multiple long-term health conditions within a single individual is increasingly common and presents a major challenge to global health systems, especially as populations age [1]. This clustering of chronic diseases contributes to higher rates of healthcare use, diminished functional capacity, complex medication regimens, and a greater risk of early death [2, 3]. Among the various forms of multimorbidity, cardiometabolic multimorbidity (CMM) has gained particular attention due to its significant clinical and public health implications. CMM typically refers to the co-occurrence of chronic conditions such as type 2 diabetes (T2D), hypertension, cardiovascular disease (CVD), coronary heart disease (CHD), and stroke within the same individual [4]. This clustering of diseases reflects shared pathophysiological mechanisms, primarily related to insulin resistance, chronic inflammation, endothelial dysfunction, and metabolic dysregulation. The burden of CMM is considerable: it is associated with substantially elevated risks of hospitalization, disability, healthcare costs, and premature death [5]. Traditional risk factors such as advancing age, physical inactivity, poor dietary habits, smoking, and obesity are well-established contributors to CMM [6]. Among these, obesity, particularly abdominal or visceral obesity, plays a pivotal etiological role [7].

Abdominal obesity, often indicative of visceral adiposity, is among the most critical and modifiable risk factors for cardiometabolic disorders [8, 9]. Historically, body mass index (BMI) and waist circumference (WC) have been widely used in both clinical and research settings to evaluate obesity and its related health risks. The associations of BMI and WC with adverse cardiometabolic outcomes are well documented [9]. However, both indices have notable limitations. BMI, while convenient and widely adopted, does not distinguish between fat mass and lean muscle mass and fails to account for the distribution of adiposity [10, 11]. Consequently, BMI may underestimate risk in individuals with a normal weight but excessive visceral fat and may paradoxically suggest protective effects in certain populations, such as those with heart failure or T2D—a phenomenon referred to as the “obesity paradox” [12]. On the other hand, WC serves as a useful surrogate for central fat accumulation but is highly correlated with weight and BMI and can be influenced by variations in body size and height, limiting its discriminatory ability in risk stratification [13]. To address these limitations, the weight-adjusted waist index (WWI) has been proposed as a more refined anthropometric measure [14]. Calculated by dividing waist circumference (WC) (in centimeters) by the square root of body weight (in kilograms), WWI aims to better reflect abdominal adiposity independent of overall body mass [14]. Unlike BMI and WC, WWI considers body composition and fat distribution and is less affected by overall body size [14]. Recent evidence has shown that WWI is independently associated with major adverse cardiometabolic outcomes, including hypertension, T2D, CVD, and all-cause mortality [15–17]. Furthermore, WWI has demonstrated superior predictive performance compared to traditional adiposity indices such as BMI and WC [18].

Despite growing interest in WWI as a novel obesity metric, evidence on its relationship with CMM remains limited. To date, only one published study has explored this association, and it was conducted exclusively within an Asian population [19]. Moreover, no research has evaluated the potential predictive utility of WWI in estimating the risk of developing CMM. This gap in the literature represents a critical barrier to fully understanding the clinical applicability of WWI in the context of multimorbidity prevention and management. Leveraging data from the English Longitudinal Study of Ageing (ELSA)—a nationally representative, prospective cohort study of middle-aged and older adults in England—offers a unique opportunity to address these evidence gaps. The current study aims to investigate the nature and magnitude of the association between WWI and the risk of CMM and to evaluate the potential utility of WWI in predicting CMM risk in this population.

## Methods

### Study population

This study was conducted following the STROBE guidelines for reporting observational epidemiological research (Supplementary Material 1) [20]. The data source for the current analysis was the ELSA, a nationally representative cohort study designed to track the health and social circumstances of individuals aged 50 years and older living in private households across England [21]. Participants in ELSA were initially drawn from earlier waves of the Health Survey for England conducted in 1998, 1999, and 2001 [21]. These respondents were subsequently invited to take part in ELSA, which officially launched in 2002–2003 with a baseline enrolment of 11,391 individuals aged 50 years and over. Since then, follow-up assessments (referred to as “waves”) have been conducted biennially [21]. For the present analysis, wave 4 (2008–2009) was selected as the baseline, and participants were followed through wave 10 (2021–2023). To construct the analytic sample, individuals with pre-existing diagnoses of hypertension, CHD, T2D, or stroke at baseline were excluded [22]. The analysis was further limited to those with complete data on WWI, relevant covariates, and cardiometabolic multimorbidity (CMM) status. After applying these eligibility criteria, a total of 3,348 participants remained in the final study population. All participants provided written informed consent, and the study protocol was approved by the London Multicentre Research Ethics Committee.

### Assessment of exposure, covariates and outcome

Sociodemographic, behavioral, and clinical data were obtained through a combination of nurse-administered interviews and structured self-completion questionnaires. These data collection tools, widely validated and routinely used in prior ELSA-based research, provided information on age, sex, physical activity levels, smoking status, alcohol consumption, and the presence of chronic conditions [21–24]. Alcohol intake was assessed by inquiring about participants’ drinking frequency over the past 12 months. Smoking history was captured in two steps: individuals were asked whether they had ever smoked, followed by their current smoking status if applicable [24]. Physical activity was self-reported via a previously validated scale that quantified engagement in light, moderate, and vigorous activities [24]. In accordance with established ELSA criteria, participants were categorized into four physical activity levels: sedentary (no moderate or vigorous activity), low (light activity only), moderate (moderate activity at least once weekly), and high (vigorous activity at least weekly) [21, 23]. During health assessments conducted by trained nurses at Mobile Examination Centres, a range of anthropometric and functional metrics were recorded. These included height, body weight, WC, handgrip strength (HGS), and blood pressure, alongside the collection of venous blood samples for biomarker analysis. Weight was measured to the nearest 0.1 kg using calibrated electronic scales, with participants wearing light indoor clothing and no shoes. Waist circumference was measured in even millimeters at the anatomical midpoint between the lowest rib and the iliac crest [25]. Body mass index was computed as weight in kilograms divided by height in meters squared (kg/m^2^), and WWI was calculated using the following formula: WWI = WC (cm) divided by the square root of body weight (kg). The WWI is a continuous indicator, with lower values indicating a healthier body composition, while higher values signal higher visceral fat accumulation and abdominal obesity. Grip strength was measured with a Smedley dynamometer (Stoelting Co., Wood Dale, IL), starting with the participant’s dominant hand. Each participant completed six alternating trials (three per hand) with a one-minute rest between attempts. The maximal value recorded across all trials was retained for analysis [26]. Blood pressure was measured in the right arm using an automated Omron HEM 907 monitor (OMRON Healthcare Europe BV, Hoofddorp, the Netherlands). Three readings were taken, and the average of the second and third was used for statistical analyses [25]. Self-reported physician diagnoses (or proxy reports when applicable) were used to ascertain the presence of chronic diseases including hypertension, CHD, T2D, and stroke.^5^ Cardiometabolic multimorbidity was operationally defined as the coexistence of two or more of the following conditions at wave 10: hypertension, CVD, diabetes, or stroke [22].

### Statistical analysis

Descriptive statistics were used to characterize the study population at baseline. Continuous variables were expressed as means and standard deviations (SD) or as medians with interquartile ranges (IQR), based on distributional assumptions verified using graphical inspection and formal tests for normality. Categorical data were summarized using counts and percentages. Comparisons between groups were performed using two-sample *t*-tests for continuous data and chi-squared tests for categorical variables. To explore whether the relationship between WWI and the likelihood of developing CMM was linear or nonlinear, we applied restricted cubic spline (RCS) logistic regression models. The spline knots were placed at the 5th, 35th, 65th, and 95th percentiles of the WWI distribution, following Harrell’s recommendations for spline modeling in moderately sized samples [27]. To determine whether nonlinearity was statistically significant, we conducted a likelihood ratio test comparing a model containing only the linear term with a model that incorporated the spline-transformed variable [28, 29]. The RCS models adjusted for a range of potential confounders including age, sex, systolic blood pressure (SBP), smoking, alcohol use, total cholesterol, high-density lipoprotein cholesterol (HDL-C), HGS, and physical activity. Subsequently, multivariable logistic regression was used to assess the association between WWI and CMM. Odds ratios (ORs) with 95% confidence intervals (CIs) were reported. To account for confounding, three models were specified with increasing levels of adjustment. Model 1 included basic demographic factors: age and sex. Model 2 added behavioral and clinical covariates: smoking status, alcohol intake, SBP, total cholesterol, HDL-C, and HGS. Model 3 further incorporated physical activity levels. The covariates were chosen based on established links with cardiometabolic health, prior findings from ELSA-based research [22, 23, 26, 30] and their potential influence on the WWI-CMM relationship [31]. Given that spline analyses suggested a mostly linear association, WWI was treated as a continuous variable in the main regression models, scaled per SD increase. As a secondary analysis, WWI was also categorized into tertiles to assess consistency of results across exposure levels. To evaluate the predictive utility of WWI for CMM, we calculated the concordance index (C-index) for model discrimination. Two logistic regression models were developed: one containing only traditional risk factors (age, sex, smoking, alcohol use, SBP, total cholesterol, HDL-C, HGS, and physical activity), and another that included WWI alongside these predictors. The change in the C-index between these models was used to quantify any improvement in predictive accuracy, with statistical significance assessed using DeLong’s test [32]. Additionally, we tested the improvement in model fit using the −2 log likelihood statistic, which is a sensitive measure of risk discrimination [33, 34]. Differences in the −2 log likelihood between models with and without WWI were calculated to determine whether the inclusion of WWI significantly enhanced prediction. All statistical analyses were performed using Stata version MP 18.0 (StataCorp, College Station, TX, USA).

## Results

Baseline characteristics of the study's participants overall and according to WWI tertiles are presented in Table 1. The overall mean (SD) age of the 3,348 study participants at baseline was 64 (9) years and males constituted 45.1% of the cohort. The mean (SD) WWI was 10.88 (0.77). Participants with higher WWI (tertile 3) were older and more likely to be male, had higher levels of anthropometric indices (BMI, WC, and weight) except for height which was lower, less likely to have moderate to high levels of physical activity or consume alcohol frequently, had higher levels of SBP and triglycerides, and lower levels of total cholesterol and HDL-C.

**Table 1:** Baseline characteristics overall and by categories of WWI

		Weight-adjusted waist index			
Characteristic	Overall (N = 3348)	Tertile 1 (N = 1116)	Tertile 2 (N = 1116)	Tertile 3 (N = 1116)	*p*-value
	Mean (SD) or Median (IQR) or n (%)	Mean (SD) or Median (IQR) or n (%)	Mean (SD) or Median (IQR) or n (%)	Mean (SD) or Median (IQR) or n (%)	
WWI	10.88 (0.77)	10.06 (0.42)	10.89 (0.17)	11.68 (0.52)	<.001
Body mass index, kg/m^2^	27.6 (4.9)	25.2 (3.8)	27.5 (4.3)	29.9 (5.3)	<.001
Waist circumference, cm	95.0 (13.3)	84.3 (9.3)	95.4 (9.2)	105.3 (11.8)	<.001
Height, cm	166.7 (9.5)	167.2 (9.5)	167.4 (9.7)	165.4 (9.2)	<.001
Weight, kg	76.7 (15.6)	70.8 (13.3)	77.4 (14.4)	82.0 (16.9)	<.001
Age, yrs	63.5 (8.5)	60.9 (7.4)	63.3 (7.8)	66.1 (9.4)	<.001
Sex					<.001
Male	1510 (45.1%)	332 (29.7%)	548 (49.1%)	630 (56.5%)	
Female	1838 (54.9%)	784 (70.3%)	568 (50.9%)	486 (43.5%)	
Current smoker					.42
No	2873 (85.8%)	953 (85.4%)	970 (86.9%)	950 (85.1%)	
Yes	475 (14.2%)	163 (14.6%)	146 (13.1%)	166 (14.9%)	
Alcohol categories					.012
None	1121 (33.5%)	337 (30.2%)	361 (32.3%)	423 (37.9%)	
1–2 times/wk	843 (25.2%)	297 (26.6%)	282 (25.3%)	264 (23.7%)	
3–4 times/wk	636 (19.0%)	226 (20.3%)	216 (19.4%)	194 (17.4%)	
5 or more times/wk	748 (22.3%)	256 (22.9%)	257 (23.0%)	235 (21.1%)	
Handgrip strength, kg	31.0 (11.4)	30.1 (10.9)	32.3 (11.8)	30.6 (11.3)	<.001
SBP, mmHg	130 (17)	126 (16)	131 (16)	135 (18)	<.001
Total cholesterol, mmol/L	5.84 (1.14)	5.93 (1.08)	5.88 (1.11)	5.70 (1.21)	<.001
HDL cholesterol, mmol/L	1.59 (0.42)	1.75 (0.44)	1.55 (0.39)	1.48 (0.38)	<.001
Triglyceride, mmol/L	1.40 (1.00, 2.00)	1.20 (0.90, 1.60)	1.50 (1.10, 2.10)	1.70 (1.20, 2.40)	<.001
PA level					<.001
Sedentary	91 (2.7%)	18 (1.6%)	27 (2.4%)	46 (4.1%)	
Low	593 (17.7%)	143 (12.8%)	163 (14.6%)	287 (25.7%)	
Moderate	1810 (54.1%)	600 (53.8%)	634 (56.8%)	576 (51.6%)	
High	854 (25.5%)	355 (31.8%)	292 (26.2%)	207 (18.5%)	

HDL, high-density lipoprotein; PA, physical activity; SBP, systolic blood pressure; SD, standard deviation; WWI, weight-adjusted waist index

**Fig. 1:**
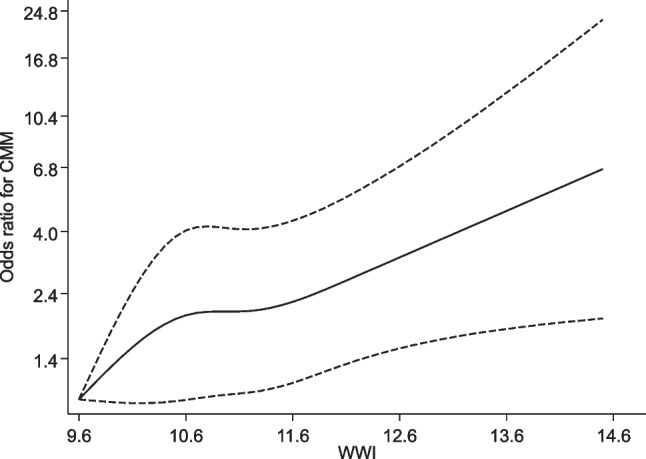
Restricted cubic spline curve of the association between WWI and risk of cardiometabolic multimorbidity. WWI, weight-adjusted waist index. Reference value for WWI is 9.6; dashed lines represent the 95% confidence intervals for the spline model (solid line). The model was adjusted for age, sex, smoking status, alcohol consumption, systolic blood pressure, total cholesterol, high density lipoprotein cholesterol, handgrip strength, and physical activity

**Fig. 2:**
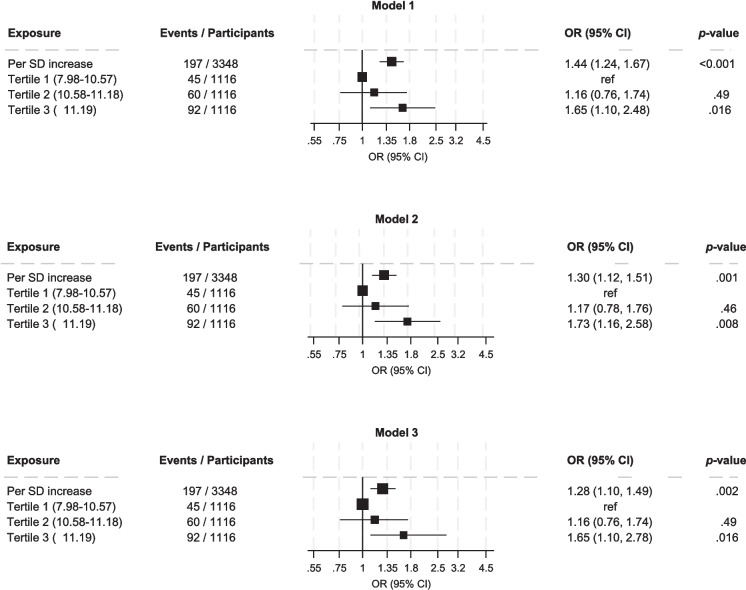
Associations of WWI with cardiometabolic multimorbidity. CI, confidence interval, OR, odds ratio; SD, standard deviation; WWI, weight-adjusted waist index. Model 1: Adjusted for age and sex. Model 2: Model 1 plus smoking status, alcohol consumption, systolic blood pressure, total cholesterol, high density lipoprotein cholesterol, and handgrip strength. Model 3: Model 2 plus physical activity

A total of 197 participants developed CMM after 15 years of follow-up. In the RCS analysis, there was evidence of linear dose–response relationship between WWI and the risk of CMM (*p* for nonlinearity = 0.44); the risk of CMM increased continuously across the WWI range 9.6 to 14.6 (Fig. 1). In analysis adjusted for age, sex, smoking status, alcohol consumption, SBP, total cholesterol, HDL-C and HGS, the OR for CMM per 1SD increase in WWI was 1.30 (95% CI: 1.12–1.51) (Fig. 2-Model 2), which was minimally attenuated following further adjustment for physical activity 1.28 (95% CI: 1.10–1.49) (Fig. 2-Model 3). Comparing the extreme tertiles of WWI, the corresponding adjusted ORs were 1.73 (95% CI: 1.16–2.58) and 1.65 (95% CI: 1.10–2.78), respectively (Fig. 2-Models 2 and 3).

Measures of risk discrimination are presented in Table 2. The CMM risk prediction model containing established risk factors yielded a C-index of 0.6892 (95% CI, 0.6500, 0.7285). The C-index was 0.6957 (95% CI, 0.6565, 0.7350) on addition of information on WWI, representing a non-significant increase of 0.0065 (*p*-value = 0.29). The −2 log likelihood was significantly improved on addition of WWI (*p* for comparison = 0.001).

**Table 2:** Measures of risk discrimination upon addition of WWI to a CMM risk model containing established risk factors

Measure of discrimination	Estimates
C-index (95% CI): established risk factors	0.6892 (0.6500, 0.7285)
C-index (95% CI): established risk factors plus WWI	0.6957 (0.6565, 0.7350)
C-index change (*p*-value)	0.0065 (.29)
*p*-value for difference in −2 log likelihood	.001

CI, confidence interval; CMM, cardiometabolic multimorbidity; WWI, weight-adjusted waist index; established risk factors include age, sex, smoking status, alcohol consumption, systolic blood pressure, total cholesterol, high-density lipoprotein cholesterol, handgrip strength, and physical activity level

## Discussion

In this representative cohort of adults aged 50 years and older, individuals in the highest tertile of WWI were generally older, more often male, had higher BMI, WC, and weight, but were shorter in stature. They also exhibited less favorable lifestyle profiles, including lower levels of physical activity, as well as adverse cardiometabolic profiles characterized by higher SBP and triglycerides, and lower HDL-C levels. A clear linear dose–response association was observed between WWI and the risk of CMM, with risk increasing steadily across WWI distribution. Multivariable analyses adjusting for both traditional and emerging risk factors (including physical activity and HGS) confirmed that higher WWI levels were independently associated with greater CMM risk. Although the addition of WWI to a conventional risk prediction model did not result in a statistically significant improvement in the C-index, it did significantly enhance model discrimination as assessed by the −2 log likelihood test.

The relationship between the WWI and adverse cardiometabolic outcomes has been explored in several studies; however, most of this evidence originates from Asian populations [15–17] or have been based on cross-sectional data, [35, 36] which limits causal inference due to the absence of temporality. To date, only one longitudinal study has specifically investigated the association between WWI and the risk of CMM. In 2024, Xia et al., using data from the Chinese Kailuan cohort, examined several adiposity indices—including BMI, WHR, WHtR, and WWI—and found that higher WWI was associated with increased risks of transitioning from a healthy state to first cardiometabolic disease (FCMD), and subsequently from FCMD to CMM [19]. Our findings build on this limited body of evidence by demonstrating, for the first time, a linear and independent association between WWI and incident CMM in a nationally representative cohort of older adults in a Western setting. Furthermore, our study adds novel insights into the predictive value of WWI, showing that while it modestly improves model discrimination beyond conventional risk factors, its contribution is statistically significant when assessed using a sensitive likelihood-based metric.

 The association between higher WWI and increased risk of CMM likely reflects the adverse metabolic consequences of excess visceral fat. Individuals with lower WWI values tend to exhibit healthier body composition, including more favorable fat distribution, lower levels of visceral adiposity, and potentially greater muscle mass—all of which contribute to better metabolic regulation. In contrast, higher WWI values signify greater abdominal obesity and visceral fat accumulation, and may also indicate sarcopenic obesity, characterized by reduced muscle mass alongside central fat excess. Visceral adiposity plays a central role in the pathogenesis of cardiometabolic conditions such as dyslipidemia, hypertension, T2D, and CVD, which together comprise CMM [37, 38]. As visceral fat expands, it promotes a state of chronic low-grade inflammation, oxidative stress, and insulin resistance—processes driven by increased secretion of pro-inflammatory cytokines and macrophage infiltration [39]. Ectopic fat deposition in key metabolic organs such as the liver, pancreas, and skeletal muscle further impairs glucose and lipid metabolism, compounding systemic dysfunction [40]. In addition, excess visceral fat is linked to reduced adiponectin levels and leptin resistance, both of which hinder insulin sensitivity and metabolic homeostasis [40, 41]. These disruptions accelerate lipolysis and elevate circulating free fatty acids and inflammatory mediators, [42] contributing to endothelial dysfunction, vascular inflammation, and pro-thrombotic states—mechanistic features that underlie the clustering of conditions that define CMM.

The findings of this study highlight the potential value of the WWI as a practical tool for identifying individuals at elevated risk of CMM. WWI is a simple, non-invasive, and cost-effective measure that can be easily calculated using routine clinical data, making it suitable for both large-scale population screening and clinical practice. Unlike traditional anthropometric indices, WWI better distinguishes fat distribution from total body mass and offers a more accurate reflection of visceral adiposity—the metabolically active fat depot most strongly linked to cardiometabolic risk [7]. Given its demonstrated association with CMM, WWI may serve as a useful adjunct to existing risk stratification approaches, particularly in settings where standard measures like BMI may be misleading, such as in older adults or individuals with T2D, where the “obesity paradox” and atypical fat distribution can obscure risk profiles. Incorporating WWI into clinical and public health risk assessments could improve early identification of at-risk individuals and inform targeted preventive strategies. Nevertheless, further research is needed to validate these findings across more diverse populations and healthcare settings, and to establish optimal WWI cut-offs that can guide clinical decision-making.

This study offers several important strengths. It is the first to examine, using a longitudinal design, the association between the WWI and the risk of CMM in a Western population, as well as the predictive value of WWI in this context. The use of data from the ELSA, a nationally representative cohort of middle-aged and older adults in England, combined with long-term follow-up, strengthens both the methodological robustness and real-world relevance of the findings. Rigorous statistical methods were applied, including extensive adjustment for conventional and novel cardiometabolic risk factors, dose–response modeling through RCSs, and assessment of predictive accuracy using validated discrimination measures. Despite these strengths, some limitations should be acknowledged. Given the observational nature of the study, causality cannot be established. Core exposures and outcomes, such as lifestyle behaviors and chronic disease status, were self-reported, which introduces the possibility of misclassification or reporting bias, including social desirability effects. While earlier research supports moderate agreement between self-reported and clinically verified diagnoses, [43, 44] the lack of medical record confirmation remains a limitation. Additionally, although a wide range of confounding variables was accounted for, residual confounding may persist, particularly due to missing data on cardiorespiratory fitness and muscle mass. Nonetheless, adjustments for physical activity (a proxy for fitness) and HGS (a reliable indicator of muscular health) help to partially address this gap. The absence of precise diagnosis dates meant time-to-event analysis could not be performed, restricting insights into the temporal sequence of associations. Furthermore, the findings may have limited generalizability to younger individuals or populations outside the UK, particularly those with different ethnic or sociodemographic characteristics. Finally, the relatively small number of CMM cases limited the ability to explore subgroup analyses.

## Conclusions

Among older adults in England, higher WWI levels were independently and linearly associated with an elevated risk of CMM. This relationship persisted even after accounting for traditional risk factors. Additionally, incorporating WWI into a standard risk model modestly improved the prediction of CMM beyond traditional risk factors, supporting its potential value in enhancing risk stratification in this population.

## Supplementary Information

Below is the link to the electronic supplementary material.

Supplementary file1 (DOCX 21 KB)

## Data Availability

Data from the ELSA are available to the public to download from the UK Data Service at https://ukdataservice.ac.uk/.
